# Unusual clinical presentations in an aquaporin-4 antibody-positive patient: a case report

**DOI:** 10.11604/pamj.2025.50.113.42228

**Published:** 2025-04-28

**Authors:** Hajar Oualmam, Mohamed Chraa, Nissrine Louhab, Najib Kissani

**Affiliations:** 1 Neuroscience Research Laboratory of Marrakech Medical School, Cadi Ayyad University, Marrakech, Morocco,; 2 Department of Neurology, Hospital Mohammed VI, Marrakech, Morocco

**Keywords:** Neuromyelitis optica, aquaporin-4 antibodies, central nervous system, case report

## Abstract

Neuromyelitis optica spectrum disorder (NMOSD) is a rare autoimmune condition affecting the central nervous system, primarily targeting the optic nerves and spinal cord. We report the case of a 52-year-old patient with no significant medical history who presented with bilateral lower limb heaviness and sensory disturbances. Neurological examination revealed peripheral neurogenic syndrome and syringomyelic dissociation. The presence of anti-aquaporin 4 (AQP4) antibodies confirmed the diagnosis of NMOSD. Treatment with corticosteroids and immunosuppressants led to clinical improvement. This case highlights the need for heightened clinical suspicion in patients with atypical neurological symptoms, as NMOSD can present beyond its classical opticospinal manifestations, ensuring timely diagnosis and appropriate management.

## Introduction

Neuromyelitis optica spectrum disorder (NMOSD) is a rare autoimmune demyelinating disease of the central nervous system (CNS) primarily affecting the optic nerves and spinal cord. It is strongly associated with autoantibodies against aquaporin-4 (AQP4), a water channel protein expressed on astrocytes, leading to astrocytopathy and secondary neuroinflammation [1]. The clinical presentation of NMOSD typically includes optic neuritis, longitudinally extensive transverse myelitis, and area postrema syndrome, but atypical manifestations can occur, often complicating diagnosis [2]. Peripheral nervous system involvement is uncommon in NMOSD and may lead to misdiagnosis or delayed treatment [3]. Early recognition and appropriate management are crucial, as untreated NMOSD can result in severe disability. We report a case of a 52-year-old patient presenting with predominant peripheral neurogenic syndrome with syringomyelic dissociation, highlighting an atypical manifestation of NMOSD.

## Patient and observation

**Patient information:** a 52-year-old patient with no significant medical history, recent vaccinations, or infectious episodes presented with progressive bilateral lower limb weakness and sensory disturbances.

**Clinical findings:** neurological examination revealed peripheral neurogenic syndrome affecting both lower limbs, with syringomyelic dissociation.

**Chronology of current episode:** one month prior to admission, the patient experienced the onset of tingling and electric-like sensations in the lower limbs. This was followed by progressive motor weakness with symmetrical ascending deficits. The patient later developed acute urinary retention and constipation. No fever or systemic symptoms were reported.

**Figure 1:**
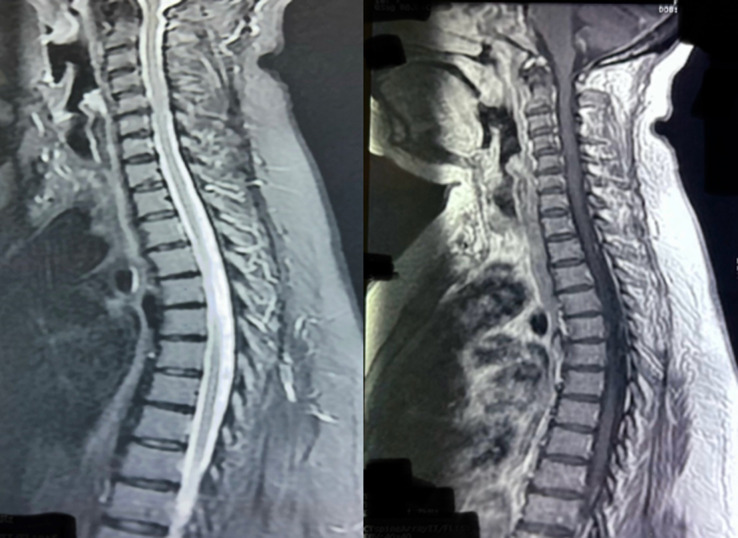
magnetic resonance imaging showing syringomyelic cavity

**Diagnostic assessment:** a lumbar puncture revealed an elevated leukocyte count (160 cells/mm^3^, 70% lymphocytes), decreased glucose levels (0.52 g/L), and increased protein levels (0.92 g/L). Cultures identified Haemophilus influenzae. Magnetic Resonance Imaging (MRI) revealed a syringomyelic cavity extending from D2 to D7 (Figure 1). Electromyography (EMG) showed sensory-motor axonal polyradiculoneuropathy. Serological, immunological, and phosphocalcic tests showed no abnormalities. Anti-AQP4 antibody testing was positive, supporting the diagnosis.

**Diagnosis:** neuromyelitis optica spectrum disorder (NMOSD) associated with syringomyelia and polyradiculoneuropathy.

**Therapeutic interventions:** the patient received intravenous ceftriaxone (2g twice daily for 21 days) for the infectious component. Immunotherapy was initiated with intravenous methylprednisolone (1g/day for 3 days), followed by long-term immunosuppressive therapy.

**Follow-up and outcome of interventions:** the patient demonstrated significant motor recovery and improvement in sensory symptoms. Ongoing follow-up in neurology is ensuring disease monitoring and symptom management.

**Patient perspective:** the patient reported noticeable improvement in mobility and relief from sensory disturbances. The patient and family expressed satisfaction with the treatment outcome.

**Informed consent:** written informed consent was obtained from the patient.

## Discussion

**Epidemiology and pathophysiology:** neuromyelitis optica spectrum disorder (NMOSD) is a rare autoimmune disease primarily affecting the optic nerves and spinal cord, characterized by the presence of anti-aquaporin-4 (AQP4) antibodies. Anti-aquaporin-4 (AQP4) is a water channel protein located on astrocyte membranes in the central nervous system. When AQP4 antibodies bind to their target, they induce a cascade of pathological events, including the activation of the complement system, antibody-dependent cellular cytotoxicity, and subsequent inflammation, demyelination, and axonal damage [2]. While NMOSD is traditionally associated with optic neuritis and longitudinally extensive transverse myelitis, its clinical manifestations can vary, posing challenges in early diagnosis and treatment.

**Differential diagnosis:** the differential diagnosis for NMOSD includes multiple sclerosis (MS) and myelin oligodendrocyte glycoprotein (MOG)-antibody-associated diseases. Although both MS and NMOSD affect the central nervous system, they have distinct pathophysiological mechanisms and different serological markers. Neuromyelitis optica spectrum disorder is typically diagnosed by the detection of AQP4 antibodies, while MS is characterized by oligoclonal bands and plaques on MRI. Additionally, MOG antibody-associated disease can present with similar neurological symptoms but has different radiological features, such as peri-neural enhancement on MRI, helping differentiate it from NMOSD [3-5]. The accurate identification of these conditions is crucial, as the treatment and prognosis vary significantly between them.

**Associations with systemic conditions:** neuromyelitis optica spectrum disorder has been reported in association with various autoimmune and infectious diseases. These include collagen vascular diseases, systemic lupus erythematosus (SLE), and infections like tuberculosis and HIV [6-9]. For instance, the literature has documented cases of NMOSD triggered by viral infections, such as varicella-zoster virus and infectious mononucleosis [10]. Although the pathophysiological link between SLE and NMOSD remains unclear, some studies have suggested that antiphospholipid antibodies, such as anticardiolipin antibodies and lupus anticoagulant, may play a role in the development of NMOSD in patients with connective tissue disorders [6]. These associations highlight the complex and multifactorial nature of NMOSD.

**Management and prognosis:** early diagnosis and treatment are essential to improving outcomes in NMOSD. Acute attacks are typically treated with high-dose corticosteroids, and long-term management often involves immunosuppressive therapy, including drugs such as rituximab, azathioprine, or mycophenolate mofetil [7]. The management strategy must be tailored to the individual patient based on their clinical presentation and disease course. Our case emphasizes the importance of considering NMOSD in patients with atypical presentations, particularly in the presence of AQP4 antibodies. Prompt recognition of the disease can lead to better control of relapses and prevention of long-term disability.

## Conclusion

This case illustrates an atypical presentation of neuromyelitis optica spectrum disorder, with peripheral neurogenic syndrome and syringomyelia in a 52-year-old patient confirmed by the presence of AQP4 antibodies. The favorable outcome after appropriate antimicrobial and immunosuppressive therapy emphasizes the importance of considering NMOSD in patients with unexplained neurological deficits, even when the presentation extends beyond classical opticospinal involvement, to ensure early diagnosis and optimal management.
